# Monophosphoryl lipid A boosts macrophage antimicrobial immunity by metabolically regulating source-specific ROS generation

**DOI:** 10.3389/fimmu.2026.1745195

**Published:** 2026-03-30

**Authors:** Dan Hao, Benjamin D. Klein, Margaret A. McBride, Julia K. Bohannon, Naeem K. Patil, Xenia D. Davis, Mary A. Oliver, Mei Lin Ning Dye, Sara Weidenbach, Jamey D. Young, Edward R. Sherwood

**Affiliations:** 1 Department of Anesthesiology, Vanderbilt University Medical Center, Nashville, TN, United States; 2 Department of Pathology, Microbiology and Immunology, Vanderbilt University Medical Center, Nashville, TN, United States; 3 East Tennessee State University, Quillen College of Medicine, Johnson, TN, United States; 4 Department of Chemical and Biomolecular Engineering, Vanderbilt University, Nashville, TN, United States; 5 Department of Molecular Physiology and Biophysics, Vanderbilt University, Nashville, TN, United States

**Keywords:** trained immunity, innate immune memory, monophosphoryl lipid A (MPLA), reactive oxygen species (ROS), NADPH oxidase (NOX), NADPH, xanthine oxidase (XO)

## Abstract

**Introduction:**

Monophosphoryl lipid A (MPLA), a toll-like receptor (TLR) 4 agonist and licensed vaccine adjuvant, reprograms innate immune cells to confer protection against diverse pathogens. However, the metabolic and molecular adaptations supporting this response remain poorly defined.

**Methods:**

The contributions of discrete reactive oxygen species (ROS) sources—including NADPH oxidase 2 (NOX2), xanthine oxidase (XO), mitochondria, and inducible nitric oxide synthase (iNOS)—to MPLA-induced macrophage antimicrobial activity were examined using genetic deletion or pharmacologic inhibition. Metabolic and redox adaptations supporting this response were assessed by analyzing oxidative pentose phosphate pathway (oxPPP) activity, glutathione-dependent antioxidant systems, and mitochondrial oxidative phosphorylation in MPLA-primed macrophages.

**Results:**

MPLA enhanced macrophage clearance of *Pseudomonas aeruginosa* by coordinating source-specific ROS generation. NOX2 was essential for this response, as its pharmacologic inhibition or genetic deletion markedly diminished MPLA-induced microbicidal responses. MPLA also induced XO, providing auxiliary ROS that acted additively with NOX2-derived ROS to support bacterial clearance. MPLA activated the oxPPP to generate NADPH, which was essential for supporting phagocytosis and maintaining glutathione-dependent redox homeostasis. Additionally, MPLA promoted mitochondrial oxidative phosphorylation to sustain phagocytic capacity. Mitochondrial ROS (mROS) were tightly constrained by induction of antioxidant systems, including superoxide dismutase 2 (SOD2), heme oxygenase-1 (HO-1) and glutathione, and were dispensable for antimicrobial protection. iNOS-derived nitric oxide did not contribute to the MPLA-induced antimicrobial phenotype.

**Conclusion:**

These findings define the metabolic and redox circuits driving MPLA-induced antimicrobial immunity and establish its potential as a host-directed antimicrobial therapy beyond vaccine adjuvancy.

## Introduction

The persistent burden of infectious diseases continues to challenge global health despite decades of antibiotic development (1, 2). This ongoing challenge underscores the need for strategies that reinforce host defense, particularly in populations that are vulnerable to infection. Emerging evidence shows that innate myeloid cells can acquire memory of previous encounters with microbe-derived ligands, enabling amplified and broad-spectrum antimicrobial responses upon subsequent infection—a phenomenon known as innate immune memory, or trained immunity (3, 4). Lipopolysaccharide (LPS), a key component of the cell wall of Gram-negative bacteria and canonical toll-like receptor (TLR) 4 agonist, has been recognized for its immunostimulatory properties since the 1950s (5), but its clinical application is limited by toxicity. Monophosphoryl lipid A (MPLA), a detoxified derivative of LPS, retains the immunomodulatory capacity of LPS but with a favorable safety profile and is approved by the Food and Drug Administration (FDA) as a vaccine adjuvant in licensed human papillomavirus (HPV), shingles and hepatitis B vaccines (6, 7).

We, and others, have shown that MPLA boosts antimicrobial immunity, conferring broad, durable protection against diverse pathogens for over two weeks (8–13). The protective phenotype is dependent on reprogramming innate myeloid cells, with macrophages playing a key role. As frontline effectors of innate immunity, macrophages eliminate pathogens through phagocytosis and facilitate antimicrobial immunity via generation of antimicrobial effectors (14, 15). Importantly, macrophage function is tightly linked to cellular energy metabolism, with distinct activation states supported by characteristic metabolic programs. Classically activated (M1) macrophages rely predominantly on aerobic glycolysis to sustain inflammatory and microbicidal functions, whereas alternatively activated (M2) macrophages preferentially engage mitochondrial oxidative phosphorylation and fatty acid oxidation to support tissue repair and resolution of inflammation. This metabolic plasticity enables macrophages to respond appropriately to distinct microenvironment signals (16, 17).

Emerging evidence indicates that trained immunity is accompanied by durable metabolic rewiring, allowing macrophages to rapidly meet the energetic and biosynthetic demands of antimicrobial defense. MPLA-primed macrophages exhibit enhanced glycolysis and oxidative phosphorylation in parallel with augmented phagocytic capacity and microbial killing (12, 18). Despite these advances, the metabolic and molecular mechanisms linking MPLA-induced metabolic reprogramming to specific antimicrobial effector pathways remain incompletely understood.

One hallmark of the MPLA-induced antimicrobial response is an amplified respiratory burst, indicative of reactive oxygen species (ROS) generation—a critical component of pathogen clearance (19, 20). Yet, the specific cellular sources of ROS and their functional relevance in MPLA-mediated innate antimicrobial responses are unknown. Furthermore, clarifying how metabolic remodeling intersects with ROS-dependent effector mechanisms is essential for understanding how MPLA reinforces innate immune defense and for identifying therapeutic strategies that leverage trained immunity without excessive inflammation.

In this study, we show that MPLA upregulates NADPH oxidase 2 (NOX2) expression, driving NOX2-dependent ROS production that is essential for macrophage-mediated clearance of *Pseudomonas aeruginosa* (*P. aeruginosa*), as both genetic deletion and pharmacological inhibition of NOX2 reduce this protection. In addition to modulating NOX2 activity, MPLA reconfigures cellular metabolism to reinforce antimicrobial function. Additionally, MPLA also increases xanthine oxidase (XO) activity to generate auxiliary ROS that act additively with NOX2-generated ROS to facilitate bacterial clearance. MPLA activates the oxidative pentose phosphate pathway (oxPPP), which supports phagocytic function and supplies NADPH to support phagocytosis and maintain glutathione-dependent redox homeostasis. Concurrently, MPLA enhances mitochondrial oxidative phosphorylation, supplying the energy required for sustained phagocytic activity. Interestingly, the MPLA-induced antimicrobial phenotype does not rely on mitochondrial ROS, which are actively modulated through induction of mitochondrial antioxidants including superoxide dismutase 2 (SOD2), heme oxygenase-1 (HO-1) and glutathione. Nitric oxide produced by the action of inducible nitric oxide synthase (iNOS) appears dispensable in this context, highlighting a selective engagement of ROS-dependent antimicrobial pathways.

## Results

### MPLA enhances *P. aeruginosa* clearance by promoting NOX2-dependent ROS production

**Figure 1:**
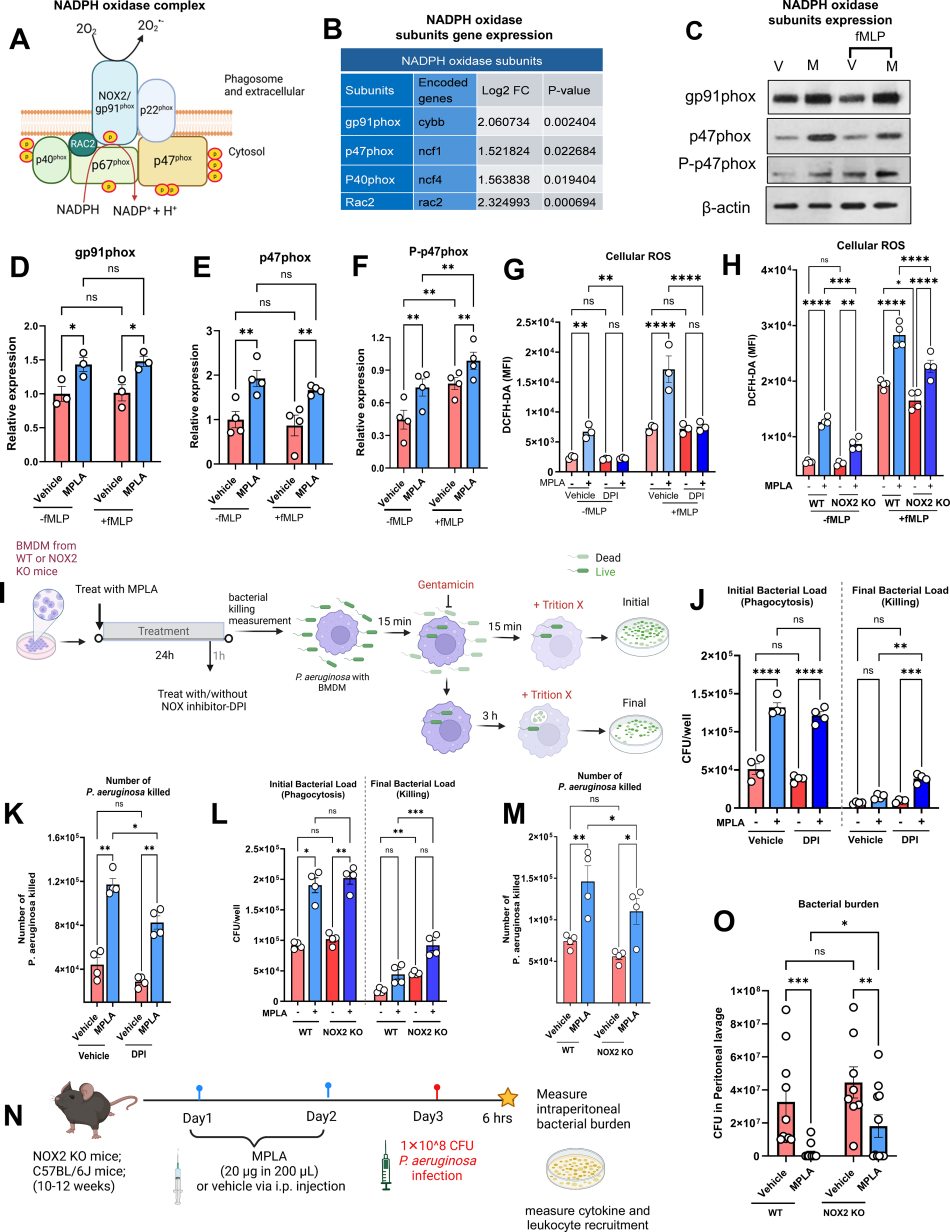
MPLA enhances *P. aeruginosa* clearance by promoting NOX2-dependent ROS production. **(A)** NADPH oxidase complex. **(B)** NOX subunit gene expression in BMDMs treated with MPLA for 24h versus vehicle. **(C)** Representative immunoblots of gp91^phox^, p47^phox^ and phosphoylated p47^phox^ (ser345) in vehicle- or MPLA- treated BMDMs with/without fMLP restimulation. **(D–F)** immunoblot quantification. **(G, H)** Cellular ROS production in BMDMs from WT or NOX2-deficient mice treated with MPLA or vehicle, with DPI or fMLP treatment as indicated. **(I)***Ex vivo* bacterial killing assay schematic. **(J–M)***P. aeruginosa* killing by MPLA-treated WT or NOX2-deficient BMDMs. **(N)***In vivo* MPLA pretreatment and infection protocol. **(O)** Peritoneal bacterial burden at 6 h post infection (n = 8-13). Data points correspond to biologically independent samples. Bars indicate mean ± s.e.m. Statistical significance was determined using two-way ANOVA. *p<0.05, **P<0.01, ***p<0.001, ****p<0.0001.

To investigate the contribution of NADPH oxidase-derived ROS to MPLA-induced antimicrobial immunity, we first assessed activation of the NADPH oxidase complex, a major source of ROS in macrophages, after MPLA treatment. This multi-subunit enzyme becomes active upon phosphorylation of cytosolic components (p40^phox^, p47^phox^, p67^phox^), which translocate to the membrane and assemble with gp91^phox^ and p22^phox^ to form the functional NOX2 complex (**Figure 1A**) (21). RNA-seq analysis in BMDMs revealed that MPLA treatment for 24 hours significantly upregulated transcripts encoding key NOX2 components, including gp91^phox^, p40^phox^, p47^phox^, Rac2 (**Figure 1B**). At the protein level, MPLA increased gp91^phox^ and p40 phox expression and strongly induced both total and phosphorylated p47^phox^ (**Figures 1C–F**; **Supplementary Figure 1**). Phosphorylation of p47^phox^ was further enhanced by co-stimulation with the formyl peptide fMLP (**Figure 1F**). Consistent with these findings, MPLA-treated macrophages exhibited a robust increase in intracellular ROS levels. This increase was abrogated in cells treated with the NOX2 inhibitor diphenyleneiodonium (DPI) and was attenuated in NOX2-deficient macrophages (**Figures 1G, H**), confirming that MPLA-induced ROS production is largely driven by NOX2.

To assess the functional significance of NOX2 activation, we performed a killing assay using *P. aeruginosa*, a clinically relevant Gram-negative pathogen. Macrophages were incubated with bacteria for 15 minutes to allow phagocytosis, followed by a 15-minute incubation with gentamicin to eliminate extracellular bacteria. Cells were then either lysed immediately and plated on tryptic soy agar to quantify viable intracellular bacteria (CFU) to assess phagocytosis or cultured for an additional 3 hours before lysis to assess killing (**Figure 1I**). MPLA treatment significantly enhanced phagocytosis of *P. aeruginosa*, as indicated by higher bacterial load at the 15-minute time point. After 3 hours, all groups showed a reduction in CFU compared to the 15-minute time point, indicating active killing of bacteria (**Figures 1J–M**). The MPLA-treated group exhibited a significantly greater reduction in viable bacteria over time compared to vehicle control, confirming enhanced intracellular killing. DPI-treated and NOX2-deficient macrophages had comparable phagocytosis at 15 minutes compared to controls but retained significantly more viable bacteria at 3 hours, indicating impaired killing capacity relative to MPLA-treated cells with intact NOX2 activity (**Figures 1J–M**).

To evaluate the contribution of NOX2 to MPLA-induced augmentation of antimicrobial immunity *in vivo*, wild-type (WT) and NOX2 knockout (KO) mice were administered MPLA on two consecutive days, followed by intraperitoneal challenge with *P. aeruginosa* 24 hours after the second MPLA treatment. Six hours post−infection, we assessed bacterial burden and leukocyte numbers in the peritoneal lavage, as well as cytokine levels in plasma (**Figure 1N**). In WT mice, MPLA significantly reduced intraperitoneal CFU of *P. aeruginosa* compared to vehicle controls (**Figure 1O**). However, MPLA-treated NOX2 KO mice exhibited approximately 10-fold higher bacterial burden compared to WT mice treated with MPLA, indicating impaired bacterial clearance in the absence of NOX2 (**Figure 1O**). MPLA treatment enhanced recruitment of innate leukocytes—including macrophages, monocytes, and neutrophils—into the peritoneal cavity in both WT and NOX2 KO mice (**Supplementary Figure 2**). MPLA treatment markedly reduced systemic inflammatory cytokine levels in both genotypes (**Supplementary Figure 3**).

Collectively, these findings demonstrate that MPLA activates the NADPH oxidase complex to drive NOX2-dependent ROS production, thereby enhancing antimicrobial capacity in macrophages.

### MPLA increases xanthine oxidase activity, generating auxiliary ROS that contributes to pathogen elimination

**Figure 2:**
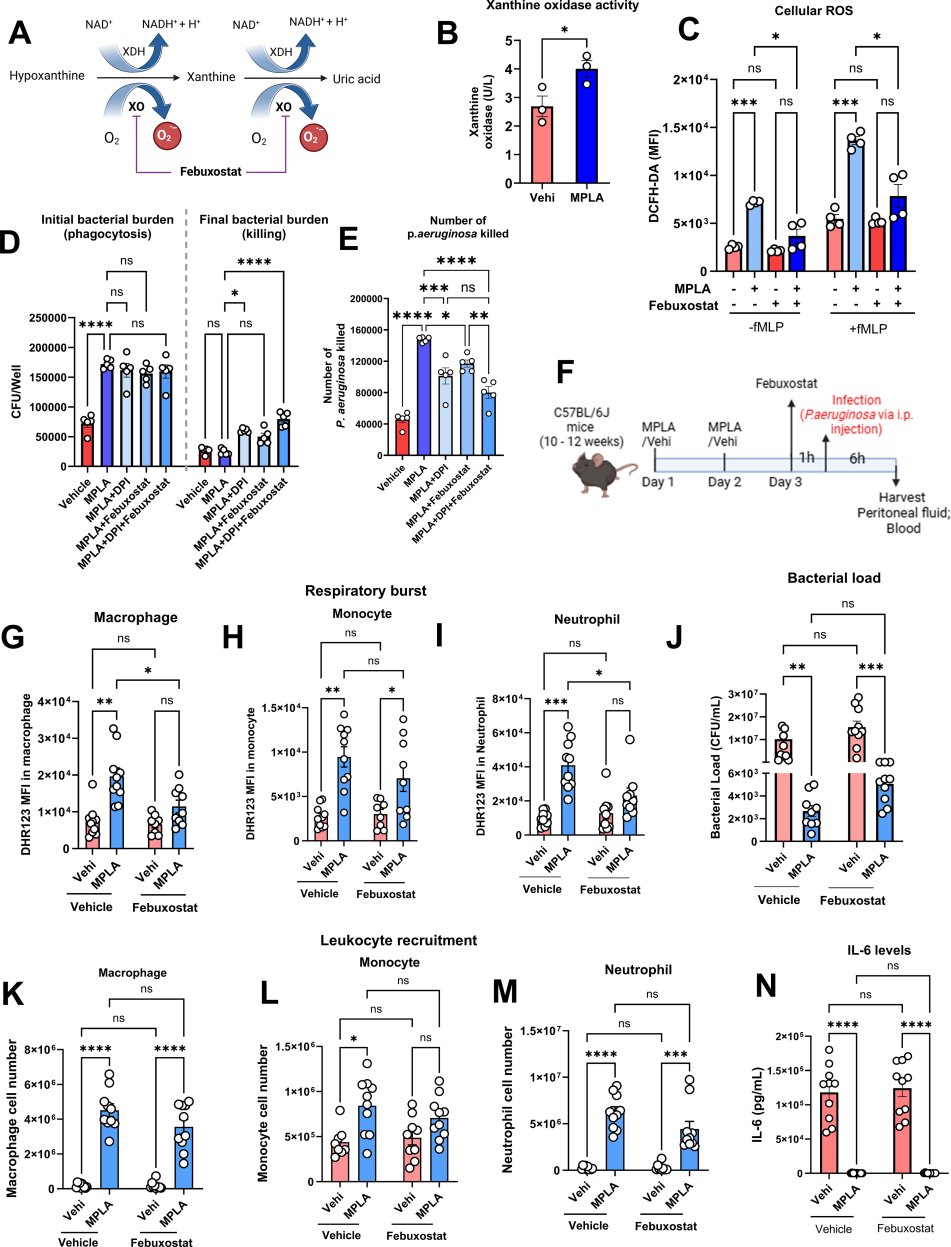
MPLA increases XO activity, generating auxiliary ROS that contribute to pathogen elimination. **(A)** Schematic of ROS generation during hypoxanthine-to-uric acid conversion by xanthine oxidase (XO). **(B)** XO activity measured at 24 h of MPLA or vehicle treatment. **(C)** Cellular ROS levels in macrophages treated with MPLA ± febuxostat (30 μM, 24 h). **(D, E)***P. aeruginosa* killing in macrophages treated with MPLA, DPI, febuxostat, and their combination. **(F)***In vivo* experiment flow. **(G–I)** Respiratory burst in macrophages, monocytes, and neutrophils. **(J)** Bacterial burden in peritoneal fluid at 6 h post-infection. **(K–M)** Leukocytes accumulation in peritoneal lavage assessed using flow cytometry **(N)**. Plasma IL-6 levels measured by ELISA (n=8 – 10). Data points correspond to biologically independent samples. Bars indicate mean ± s.e.m. Statistical significance was determined using two-tailed Students’ t test **(B)** and two-way ANOVA (others). *p<0.05, **P<0.01, ***p<0.001, ****p<0.0001.

XO generates superoxide (O_2_·^−^) and hydrogen peroxide (H_2_O_2_) by oxidizing hypoxanthine and xanthine, making it a key enzymatic ROS source in mammalian cells (**Figure 2A**) (22). To investigate the contribution of XO as a source of ROS supporting MPLA-induced antimicrobial activity, we evaluated XO activity and found it to be significantly increased in MPLA treated macrophages (**Figure 2B**). Pharmacological inhibition of XO with febuxostat, a selective non-purine inhibitor, markedly reduced MPLA-induced ROS production in BMDMs (**Figure 2C**). In bacterial killing assays, febuxostat treatment impaired killing of *P. aeruginosa*, highlighting XO-dependent ROS as a contributor to MPLA-mediated antimicrobial activity (**Figures 2D, E**). We also performed combined inhibition of XO and NOX2, and the results showed that dual inhibition had an additive effect on the bacterial killing capacity of macrophages (**Figures 2D, E**).

To assess the *in vivo* relevance of XO in MPLA-mediated host defense, mice received MPLA for two consecutive days, followed by febuxostat one hour prior to *P. aeruginosa* infection (**Figure 2F**). At 6 h post-infection, MPLA treated group exhibited significantly enhanced respiratory burst across macrophages, monocytes, and neutrophils, whereas febuxostat administration selectively reduced respiratory burst in macrophages and neutrophils, with a minimal effect in monocytes (**Figures 2G–I**). Notably, febuxostat treatment did not significantly alter MPLA-induced clearance of *P. aeruginosa*, leukocyte recruitment or suppression of IL-6 production (**Figures 2J–N**). These findings suggest that XO is a key enzymatic source of MPLA-induced ROS, playing a partial, yet functionally relevant, role in supporting cellular antimicrobial responses.

### MPLA activates the oxidative pentose phosphate pathway to support phagocytic function and sustain redox homeostasis through NADPH generation

**Figure 3:**
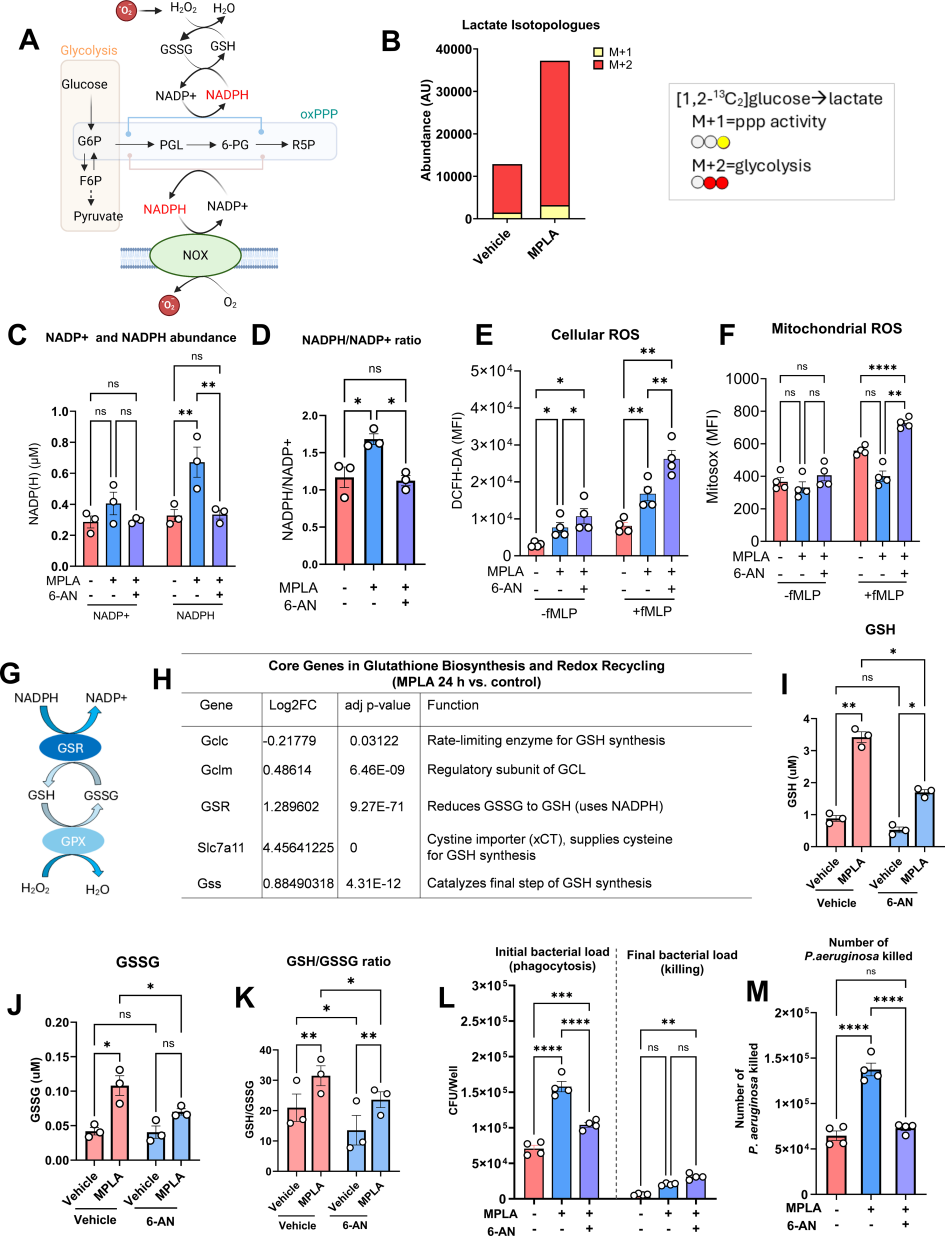
MPLA activates the oxPPP pathway to support phagocytic function and sustain redox homeostasis through NADPH generation. **(A)** Schematic of oxPPP-derived NADPH supporting NOX-dependent ROS production. **(B)** Lactate isotopologues analysis in BMDMs cultured with [1,2-¹³C_2_]glucose for 24 h **(C, D)** NADP+, NADPH levels, and NADPH/NADP+ ratios in BMDMs treated with MPLA ± 6-AN (50 μM). **(E, F)** Cellular and mROS in BMDMs following MPLA ± 6-AN treatment. **(G)** Schematic illustrating NADPH-dependent maintenance of GSH. **(H)** Expression of GSH regulatory gene after 24 h MPLA treatment. **(I–K)** GSH, GSSG, and reduced GSH to GSSG ratio in BMDMs treated with MPLA ± 6-AN. **(L, M)***P. aeruginosa* killing by MPLA-treated BMDMs in the presence or absence of 6-AN. Data represent biologically independent samples, Bars indicate mean ± s.e.m. Statistical significance was determined using one-way ANOVA **(D, L, M)** or two-way ANOVA followed with Tukey’s *post hoc* test (others). *p<0.05, **P<0.01, ***p<0.001, ****p<0.0001.

The oxPPP generates NADPH to support both NADPH oxidase-mediated ROS generation and glutathione-dependent antioxidant defense (**Figure 3A**) (23). To assess oxPPP activation, we performed isotope-tracing metabolic flux analysis by culturing BMDMs in medium supplemented with [1,2-^13^C_2_]-glucose. MPLA treatment significantly increased total lactate accumulation, predominantly as M + 2 isotopologues, with a smaller M + 1 fraction (**Figure 3B**). M + 2 labeling reflects glycolytic conversion of labeled glucose, while M + 1 enrichment indicates oxPPP flux, where the C1 carbon is released as CO_2_ (24). This finding indicates a measurable activation of oxPPP activity, which was further supported by elevated levels of NADPH and NADPH/NADP^+^ ratio (**Figures 3C, D**). Inhibition of oxPPP with 6-aminonicotinamide (6-AN), a selective inhibitor of glucose-6-phosphate dehydrogenase, significantly attenuated NADPH levels, confirming the involvement of oxPPP (**Figure 3C**).

Unexpectedly, oxPPP inhibition with 6-AN resulted in increased mitochondrial and total cellular ROS in MPLA-treated macrophages (**Figures 3E, F**). Considering the role of NADPH in supporting the glutathione antioxidant system (**Figure 3G**), we therefore assessed the effect of MPLA treatment on transcriptional profiles of key glutathione regulatory genes. This revealed upregulation of *Gsr, Slc7a11, Gss*, and *Gclm*, alongside a modest downregulation of *Gclc*, indicative of enhanced glutathione recycling and synthesis (**Figure 3H**). Consistent with these transcriptional changes, intracellular levels of reduced glutathione (GSH), oxidized glutathione (GSSG), and the GSH/GSSG ratio were markedly elevated in macrophages treated with MPLA, reflecting enhanced antioxidant capacity (**Figures 3I–K**). This elevation was significantly blunted following treatment with 6-AN, underscoring the essential role of oxPPP-derived NADPH in preserving antioxidant defense and redox homeostasis (**Figures 3I–K**).

Functionally, oxPPP inhibition reduced phagocytosis and killing of *P. aeruginosa* in MPLA-primed macrophages (**Figures 3L, M**). Collectively, these findings demonstrate that MPLA-induced metabolic reprogramming involves upregulation of oxPPP activity, which sustains NADPH production. This NADPH supply is essential for maintaining redox homeostasis and supporting antimicrobial activity beyond its role as a NOX2 substrate for ROS generation.

### MPLA enhances phagocytic activity through oxidative phosphorylation–driven bioenergetic support, independent of mitochondrial ROS production

**Figure 4:**
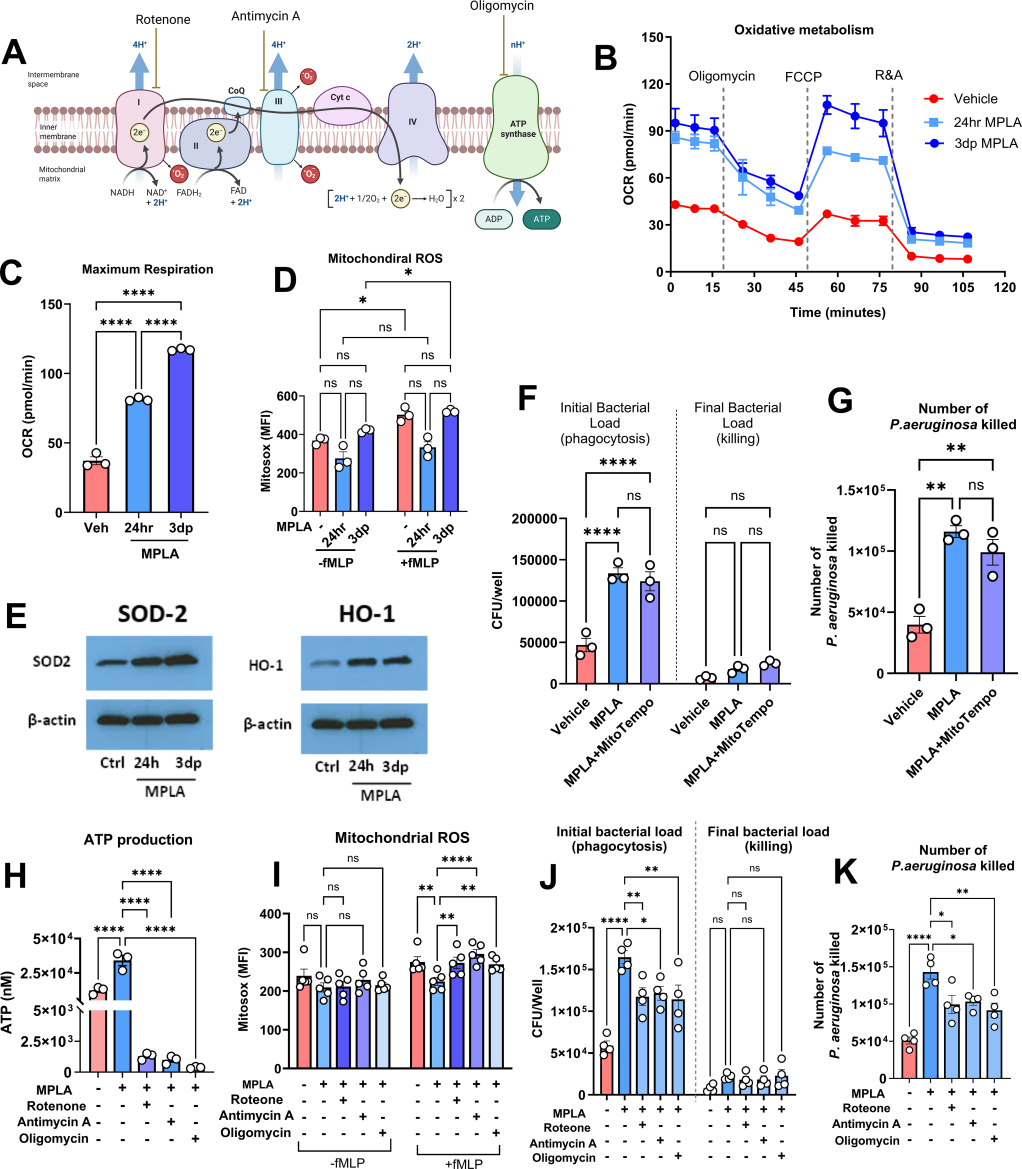
MPLA enhances phagocytic activity through oxidative phosphorylation–driven bioenergetic support, independent of mitochondrial ROS production. **(A)** Schematic of mitochondrial ATP and ROS production sites and inhibitors used. **(B)** Seahose analysis of oxygen consumption rate (OCR) in macrophages treated with MPLA for 24 h or 3 days post-treatment. **(C)** Quantification of maximal respiration. **(D)** Mitochondrial ROS levels following MPLA treatment. **(E)** Western blot analysis of SOD2 and HO-1 expression. **(F-G)***P. aeruginosa* killing by MPLA-treated BMDMs with or without MitoTEMPO. **(H–K)** Effects of mitochondrial inhibitors (rotenone, antimycin A, oligomycin) on ATP production **(H)**, mitochondrial ROS **(I)**, and bacterial killing **(J, K)** in MPLA-treated BMDMs. Data points correspond to biologically independent samples. Bars indicate mean ± s.e.m. Statistical significance was determined using one-way ANOVA **(C, F–H, J, K)**, or two-way ANOVA **(D, I)**. *p<0.05, **P<0.01, ***p<0.001, ****p<0.0001.

Mitochondrial oxidative phosphorylation (OXPHOS), driven by electron transfer through the respiratory chain, is a major contributor to ATP synthesis and ROS generation, positing it as a potential contributor to MPLA-enhanced bacterial clearance (**Figure 4A**). To assess OXPHOS activity in response to MPLA, we performed a Seahorse Mito Stress Test. MPLA stimulation elicited a robust increase in oxidative metabolism at 24 hours, with further enhancement observed at 3 days post-treatment (**Figures 4B, C**). Despite this elevated oxidative metabolism, quantification of mitochondrial ROS using MitoSOX red fluorescence showed a trend toward lower basal mROS levels at 24 hours following MPLA exposure, which rebounded by 3 days-post treatment (**Figure 4D**). Concomitantly, the mitochondrial antioxidant enzymes superoxide dismutase (SOD2) and heme oxygenase-1 (HO-1) were elevated, suggesting an adaptive mitochondrial oxidative stress response (**Figure 4E**).

To investigate the functional relevance of this mitochondrial redox adaptation in MPLA-induced antimicrobial activity, BMDMs were then treated with the mitochondria-targeted ROS scavenger MitoTEMPO. Notably, MitoTEMPO administration did not impair the enhanced bacterial killing capacity conferred by MPLA, indicating that mROS are dispensable for this effect (**Figures 4F, G**). To further assess the role of mitochondrial function, BMDMs were pretreated with MPLA for 24 hours and subsequently exposed to mitochondrial inhibitors targeting complex I (rotenone), complex III (antimycin A), and ATP synthase (oligomycin) for 1 h. These inhibitors significantly suppressed MPLA-induced ATP production (**Figure 4H**). Interestingly, complex I and III inhibition led to increased mROS levels upon fMLP re-stimulation, likely due to impaired electron transport and subsequent electron leakage to molecular oxygen (**Figure 4I**). Despite this increase in mROS, phagocytic capacity and killing of *P. aeruginosa* were markedly reduced (**Figures 4J, K**). Collectively, these findings indicate that MPLA augments macrophage antimicrobial activity through mitochondrial bioenergetic support rather than mROS generation.

### MPLA-induced NOS2-derived NO production is dispensable for bacterial clearance

**Figure 5:**
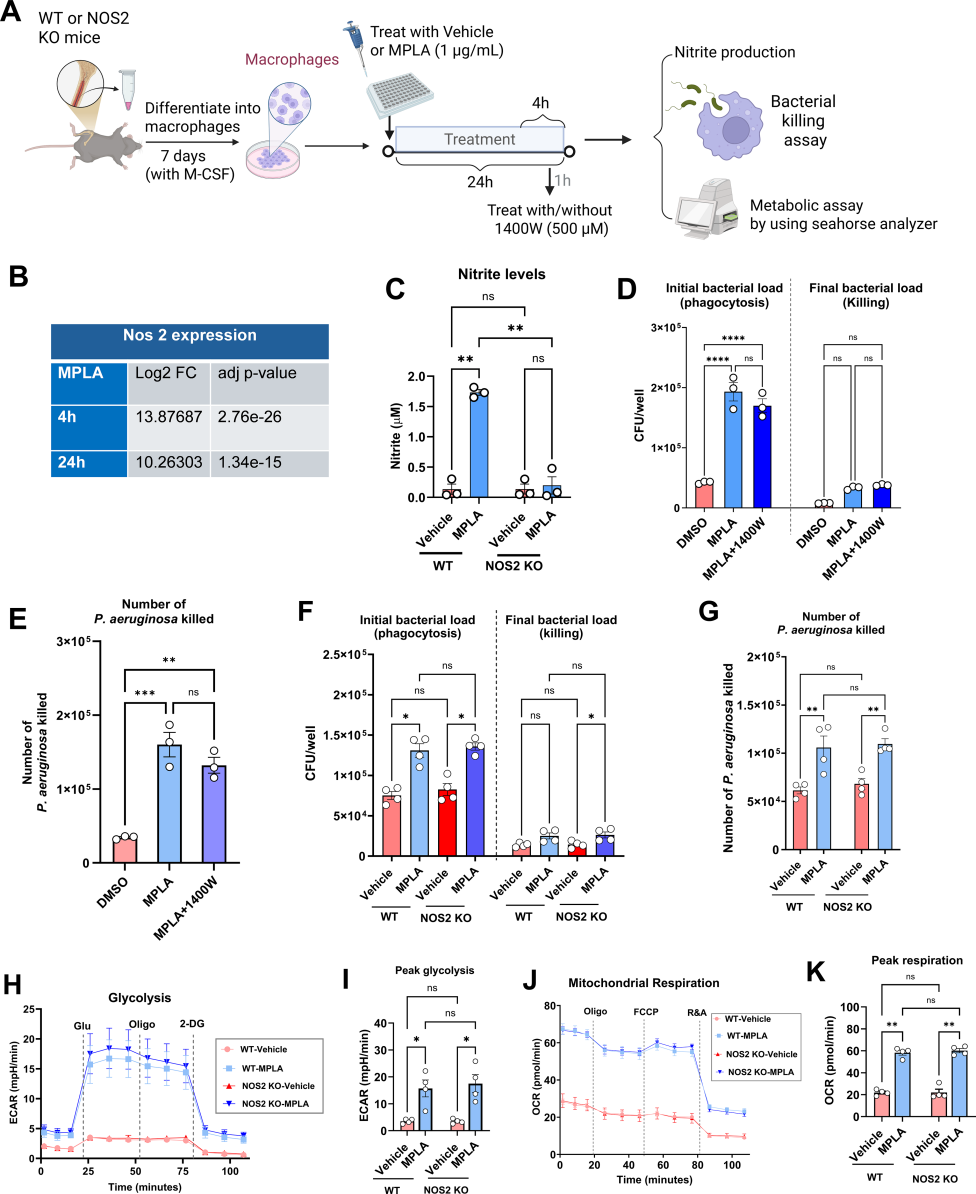
MPLA-induced NOS2-derived NO production is dispensable for bacterial clearance. **(A)** Experimental workflow **(B)** NOS2 expression analyzed by RNA-seq in BMDMs treated with MPLA for 4 or 24 hours. **(C)** Nitrite production in WT and NOS2 KO BMDMs after 24 h MPLA or vehicle treatment. **(D, E)***P. aeruginosa* killing assay in BMDMs treated with MPLA ± 1400W. **(F, G)** Bacterial killing assay in BMDMs derived from WT and NOS KO mice following 24-hour MPLA exposure. **(H, I)** Glycolytic function assessed via extracellular acidification rate (ECAR) and peak glycolytic activity. **(J, K)** Mitochondrial respiration evaluated by oxygen consumption rate (OCR) and maximal respiratory capacity. Data points correspond to biologically independent samples. Bars indicate mean ± s.e.m. Statistical significance was determined using one-way ANOVA **(C, D)**, and two-way ANOVA (others). *p<0.05, **P<0.01, ***p<0.001, ****p<0.0001.

Macrophages produce NO via NOS2 (25). To delineate the contribution of NOS2-derived NO to MPLA-induced macrophage antimicrobial function and metabolic remodeling, we performed comparative analyses using BMDMs from NOS2 KO mice alongside treatment of WT macrophages with the potent, selective NOS2 inhibitor 1400W (**Figure 5A**). MPLA stimulation elicited a robust transcriptional upregulation of NOS2, exceeding 10 log2-fold at 4 and 24 hours post-treatment (**Figure 5B**), concomitant with a significant ninefold increase in nitrite accumulation—a stable NO metabolite, at 24 hours (**Figure 5C**). Pharmacological inhibition of NOS2 with 1400W did not reduce MPLA-enhanced bactericidal activity against *P. aeruginosa* (**Figures 5D, E**). Likewise, NOS2 knockout BMDMs demonstrated complete abrogation of MPLA-driven nitrite synthesis (**Figure 5C**), yet this did not impair MPLA-enhanced bactericidal activity (**Figures 5F, G**). Considering the well-established role of metabolic reprogramming in macrophage activation phenotypes, we evaluated the impact of NOS2 deficiency on MPLA-induced metabolic shifts by Seahorse extracellular flux analysis. Notably, the enhancement of glycolytic flux and oxidative phosphorylation following MPLA exposure remained unaltered in NOS2-deficient macrophages (**Figures 5H–K**). These comprehensive data indicate that although MPLA potently induces NOS2 expression and NO production, NOS2-derived NO is dispensable for MPLA-mediated augmentation of antimicrobial effector functions and associated metabolic programming in macrophages.

## Discussion

**Figure 6:**
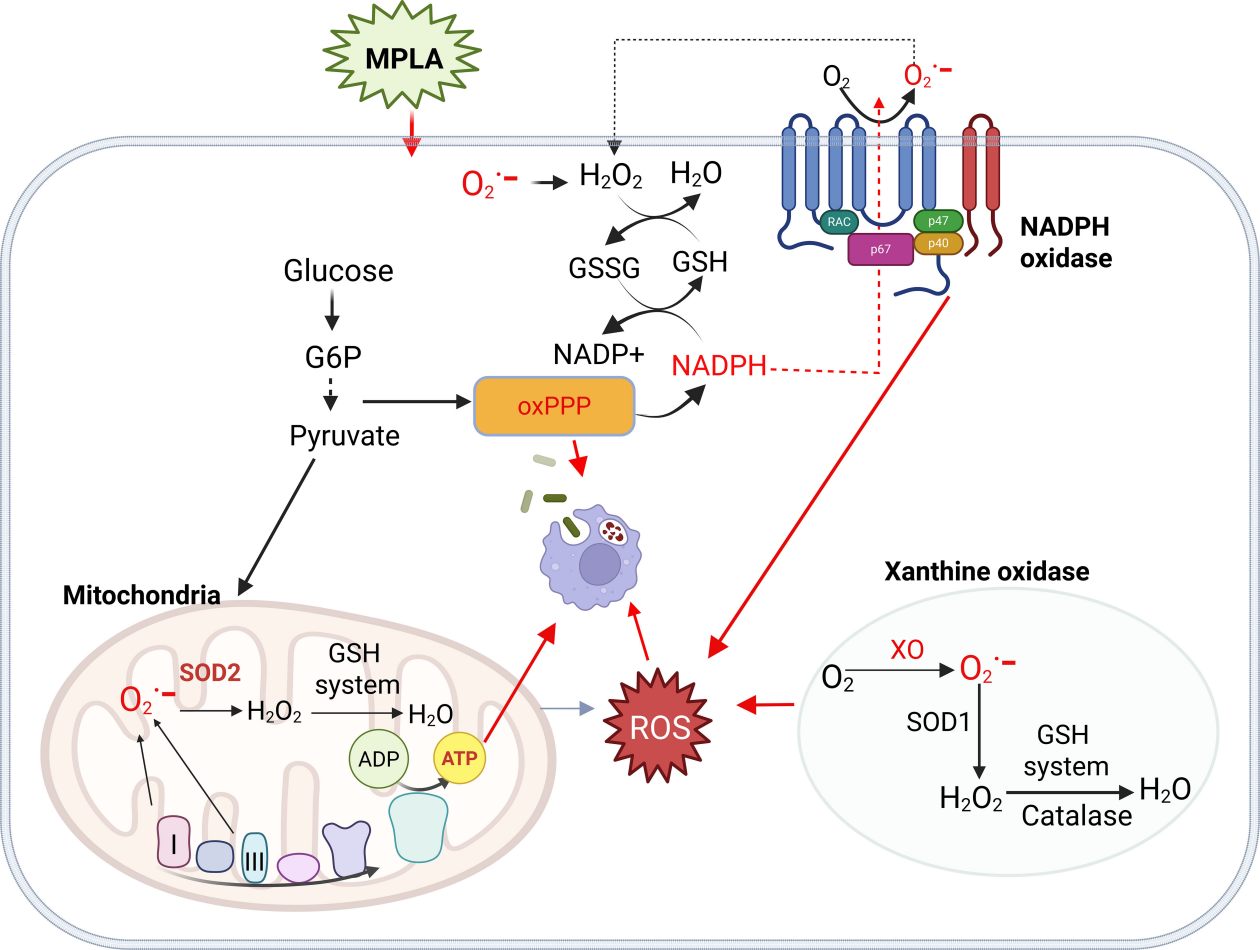
Schematic overview of key findings. MPLA enhances macrophage antimicrobial activity by promoting NOX2-dependent ROS production. It activates oxPPP to support microbial clearance and generate NADPH for redox balance via GSH/GSSG cycling. MPLA boosts mitochondrial oxidative phosphorylation to sustain bioenergetic synthesis and fuel antimicrobial function. While mitochondrial ROS are constrained by antioxidant defenses, XO provides auxiliary ROS that contribute to bacterial clearance.

Stimulation with TLR agonists and other pathogen-associated molecular patterns can prime innate immune cells for enhanced responses to infection. Despite the therapeutic potential of these alterations, the mechanisms underlying these adaptations are incompletely defined (3, 4). Here we demonstrate that NADPH oxidase- and XO-dependent ROS production are critical effectors for intracellular killing of bacteria by MPLA-primed macrophages. Further, we show that mitochondrial ROS are tightly constrained by antioxidant systems, yet MPLA boosts antimicrobial activity by enhancing mitochondrial oxidative phosphorylation and ATP generation, independent of mitochondrial ROS. In addition, MPLA activates the oxPPP, supporting phagocytosis and supplying NADPH to maintain glutathione-dependent redox homeostasis. Although NO induction is robust after MPLA treatment, it contributes minimally to bacterial clearance (**Figure 6**). Collectively, these findings define the source-specific contributions of ROS and antioxidant mechanisms in MPLA-driven innate antimicrobial immunity, positing it as a potential target for host-directed antimicrobial therapies.

MPLA primes phagocyte NADPH oxidase, leading to elevated NOX-derived ROS production and enhanced bacterial clearance. The indispensable role of this enzyme complex in host defense is well established by chronic granulomatous disease (CGD), where mutations in NOX subunits, such as gp91^phox^, p47^phox^, p22^phox^, or p67^phox^ cause recurrent infections and reduced survival (26, 27). Whether NOX activity can be inducibly regulated by a training or priming stimulus to enhance antimicrobial memory remain unclear. Our findings reveal that MPLA upregulates NOX2 expression and demonstrates that NOX2 is critical for MPLA-induced innate antimicrobial memory against *P. aeruginosa* infection.

The role of XO has been established in oxidative metabolism and vascular function (28, 29). Emerging evidence implicates XO in inflammasome activation and cytokine regulation (30). Its contribution to ROS-dependent antimicrobial activity, particularly in primed macrophages, however, remains poorly understood. Here, we show that MPLA markedly increases xanthine oxidase activity in macrophages, enhancing ROS production and partially supporting phagocytosis. Moreover, combined inhibition of XO and NOX2 produces an additive defect in bacterial killing, indicating that XO-generated ROS complement NOX2-mediated oxidative mechanisms. Collectively, these findings identify XO as a pivotal enzymatic source of ROS in innate immune priming and highlight its potential as a target to boost macrophage antimicrobial function.

The oxPPP has been shown to directly fuel NADPH oxidase-dependent ROS production and microbial killing in neutrophils (31, 32). In macrophages, we show that MPLA-induced metabolic rewiring upregulates the oxPPP, supporting phagocytosis and cellular redox buffering through NADPH generation. This could potentially influencing redox-regulated pathways such as SLC7A11-dependent cystine import, which support glutathione synthesis and cellular antioxidant capacity. Consistent with this, MPLA increased Slc7a11 expression (log_2_FC=4.6) and reduced glutathione levels, which were attenuated by oxPPP inhibition.

Functionally, treatment with the oxPPP inhibitor 6-AN significantly impaired MPLA-enhanced phagocytosis and number of *P. aeruginosa* killed. Mechanistically, phagocytosis depends on membrane expansion, fluidity, and cytoskeletal organization, processes that are supported by *de novo* fatty acid synthesis (33). Evidence showed that fatty acid synthesis is markedly upregulated during differentiation of primary human monocytes, and its inhibition reduces macrophage phagocytosis, which can be fully rescued by supplementation with a fatty acid-CoA mixture (34). Fatty acid composition further influences phagocytosis, with unsaturated fatty acids (e.g., linoleic acid, arachidate) enhancing and saturated fatty acids (e.g., palmitate) impairing phagocytic capacity (33). NADPH, as the principal reducing equivalent for fatty acid synthesis and elongation, is central to these lipid anabolic processes (35, 36). However, whether MPLA-induced metabolic reprogramming regulates macrophage fatty acid synthesis and elongation, or whether these processes depend on NADPH availability from oxidative pentose phosphate pathway to support phagocytic function, has yet to be established.

While mitochondrial ROS (mROS) have been implicated as critical mediators of LPS-induced bactericidal activity in macrophages (37, 38), our findings demonstrate that MPLA-induced antimicrobial responses occur independently of mROS. LPS activates both MyD88- and TRIF-dependent pathways, leading to TRAF6 recruitment to mitochondria and enhances mROS production (37). In contrast, MPLA primarily signals through MyD88-dependent to establish innate immune memory (39), and notably upregulates mitochondrial antioxidant enzymes such as SOD2, HO-1. Inhibition of mitochondrial complexes I and III disrupted mitochondrial bioenergetics and diminished MPLA-driven bacterial clearance, despite elevated mROS upon fMLP re-stimulation. This highlights the importance of mitochondrial bioenergetic integrity—rather than mROS generation alone—in facilitating effective bacterial killing in MPLA-primed macrophages. Collectively, these findings suggest that mROS are not universally required for macrophage bactericidal activity and highlight how pathway-specific metabolic regulation shapes distinct antimicrobial responses.

iNOS-derived NO is classically recognized as a critical effector for microbial killing in murine macrophages, and capable of forming cytotoxic peroxynitrite through rapid reaction with superoxide (40, 41). Upon MPLA stimulation, *Nos2* gene expression are upregulated by approximately 10 log2-fold, accompanied by a ninefold rise in nitrite accumulation, a stable NO metabolite. However, functional inhibition of iNOS reveals that this surge in NO contribute minimally to the enhanced bacterial clearance. These findings suggest that MPLA reprograms macrophage antimicrobial function through alternative, NO-independent mechanisms, likely dominated by ROS-mediated oxidative stress and metabolic reprogramming, rather than canonial nitrogen-centered cytotoxic pathways.

Despite significant global efforts to combat life-threatening infectious diseases, they continue to account for one in four deaths worldwide and remain major drivers of long-term disability due to infection-induced tissue damage. Host-directed therapies targeting host immune and inflammatory pathways offer a promising strategy to enhance antimicrobial defense while mitigating immunopathology. Our study delineates mechanisms by which MPLA augments innate host resistance beyond its established role as a vaccine adjuvant. We identify a critical connection between cellular metabolism and innate immune memory, showing that distinct sources of ROS contribute differentially to antimicrobial function during infection. These insights advance our understanding of the metabolic mechanisms underlying TLR4 agonist–induced innate immune training. Furthermore, the observed role of the oxidative pentose phosphate pathway in regulating NADPH-dependent antioxidant balance during MPLA treatment suggests a potential contribution to protection against infection-induced organ injury. This possibility warrants future investigation and may inform the development of strategies aimed at improving host resilience to infection and tissue damage. By defining how metabolic pathways shape robust immune responses, our findings may inform the development of novel therapeutic strategies that harness trained immunity to enhance host defense, particularly in populations with heightened susceptibility to infection.

### Limitations

Several limitations should be considered when interpreting our findings. Firstly, we did not directly assess the mechanisms by which MPLA-induced activation of the oxPPP supports phagocytosis. Fatty acid synthesis and cell membrane lipid composition are critical determinants of phagocytic capacity and are likely supported by NADPH generated via the oxPPP (33). It remains to be defined whether MPLA-induced metabolic reprogramming regulates fatty acid synthesis and elongation, and whether these processes rely on NADPH generated via the oxPPP to support macrophage phagocytic function. Second, although our study delineates MPLA-induced, macrophage-intrinsic ROS programs, we did not examine the potential influence of *P. aeruginosa*–derived metabolites such as 2-heptyl-4-hydroxyquinoline N-oxide (HQNO), pseudomonas quinolone signal (PQS), pyocyanin, and kynurenine. HQNO disrupts electron flow at the cytochrome bc1 complex, generating ROS (42), while PQS inhibits Complex I of the respiratory chain (43). Pyocyanin, a redox-active virulence factor, rapidly induces oxidative stress (44), whereas kynurenine acts as a scavenger of hydrogen peroxide and superoxide (45). How these pathogen-derived factors intersect with MPLA-driven ROS signaling during infection warrants further investigation. Finally, we did not examine how macrophages metabolize or process MPLA. Previous studies demonstrated that MPLA-induced resistance to infection can persist for at least 15 days (14). Like other lipid A derivatives, MPLA can be enzymatically modified by host phosphatases and lipases, including dephosphorylation and deacylation, which alter lipid A bioactivity (46–48). Following uptake, MPLA may be retained within endosomal or phagosomal compartments, where ligand retention and trafficking dynamics shape the balance and duration of MyD88- and TRIF-dependent TLR4 signaling (46, 49, 50). Such compartmentalized receptor engagement and intracellular processing may contribute to sustained or qualitatively altered activation states that support long-term innate immune reprogramming, which remain to be experimentally determined.

## Methods

### Mice

Male and female wild-type (C57BL/6J), NOX2 knockout (B6.129S-*Cybb^tm1Din^*/J; IMSR_JAX:002365) and NOS2 knockout (B6.129P2-*Nos2^tm1Lau^*/J; IMSR_JAX:002609) mice aged 10 to 12 weeks were purchased from the Jackson Laboratory (Bar Harbor, Maine). All experimental protocols followed the National Institutes of Health Guide for the Care and Use of Laboratory Animals and were approved by the Vanderbilt University Institutional Animal Care and Use Committee.

### Monophosphoryl lipid A

MPLA from *Salmonella enterica* serotype Minnesota Re 595 (Invivogen) was dissolved in DMSO (1 mg/mL, sonicated at 40 °C for 1 hour). MPLA was diluted 1:10 in Lactated Ringer’s solution for injection. DMSO was diluted in an identical manner as vehicle control. For cell culture experiments, MPLA was diluted to a concentration of 1 μg/ml with diluted DMSO serving as vehicle control.

### Mouse model of *P. aeruginosa* infection

Mice were treated with MPLA (20 μg in 200 μL, IP) or vehicle for 2 consecutive days prior to infection. 24 hours after the second injection of MPLA or vehicle, mice were infected with 1 ×10^8^ colony-forming units (CFUs) of *P. aeruginosa* (ATCC 19660) in 0.5 mL sterile saline via the intraperitoneal (IP) route. In some experiments, the xanthine oxidase inhibitor febuxostat (Sigma, SML1285, 200 μL 2.5 mM IP) was injected 1 hour before infection.

Six hours post-infection, rectal temperature was recorded. Whole blood was collected via carotid artery laceration under isoflurane anesthesia, and plasma was collected from blood by centrifugation (2000 g, 20 min, 4 °C) for cytokine analysis. Following cervical dislocation under anesthesia, peritoneal lavage was performed with 5 mL of cold sterile phosphate-buffered saline (PBS). A portion of lavage fluid was serial diluted, plated on tryptic soy agar, and incubated at 37 °C overnight, and bacterial colonies were counted to determine CFUs/mL of peritoneal lavage fluid. The remaining peritoneal lavage fluid was centrifuged at 300 g for 6 min at 4 °C, and the cell pellet was resuspended in PBS for flow cytometric analyses.

### Isolation, culture, stimulation and inhibitor treatment of bone marrow-derived macrophages

Bone marrow cells were flushed from mouse femurs and cultured in RPMI-1640 medium containing 2 mM glutamine, 25 mM HEPES, 10% FBS, 1% antibiotic-antimycotic (Gibco), and 10 ng/mL mouse recombinant macrophage colony stimulating factor (M-CSF, R&D Systems). Cells were differentiated into macrophages for 7 days (8, 51).

Cells were treated with MPLA (1 *μ*g/mL) or vehicle for 24 hours. In a parallel group, macrophages were treated with MPLA or vehicle followed by washout of culture medium and incubation in fresh unsupplemented media for 3 days (3dp) to induce the trained phenotype. Inhibitor treatments were applied prior to assay as follows: 6-aminonicotinamide (6-AN, Sigma, A68203) at 50 μM for 24h (52, 53); diphenyleneiodonium chloride (DPI, Sigma, D2926) at 1 μM for 1 h prior to assay (54); MitoTEMPO (Sigma, SML0737) at 500 μM for 24 h; 1400 W (Bio-techne, R&D, 1415) at 50 μM for 24 h; febuxostat (Sigma, SML1285) at 30 μM for 24 h; rotenone (Sigma, 557368) and antimycin A (Sigma, A8674) at 0.5 μM, and oligomycin (Sigma, O4876) treated at 1 μM, one hour prior to assay.

### Flow cytometry

Cells from peritoneal lavage (1 ×10^7^ cells/mL) were blocked with 1 *μ*g/mL anti-mouse CD16/32 (eBioscience), and stained with anti-F4/80-FITC (BM8), anti-Ly6G-PE (1A8), and anti-Ly6C-PE Cy5.5 (0.5 μg/10^6^ cells/0.1 mL, 15 min, RT). Neutrophils were defined as Ly6G^+^F4/80^−^, macrophages as Ly6C^−^F4/80^+^, and monocytes as Ly6C^+^F4/80^+^. Samples were acquired on a BD Accuri C6 Plus and analyzed using Accuri software.

### Cytokine and chemokine analysis

IL-6 was measured with an ELISA kit (Invitrogen, 88-7064-22). Multiple cytokines were measured using the Bio-Plex Multiplex Assay (Bio-Rad) on a Magpix Multiplex reader and analyzed with Bio-Plex Manager v6.1.

### Metabolic analysis

Cells (4 ×10^4^/well) were seeded in Seahorse XF96 plates and analyzed using an XFe96 Extracellular Flux Analyzer (Agilent). The glycolysis stress test included sequential injections of 10 mM glucose (RPI, Mount Prospect, IL), 1 μM oligomycin (Agilent Technologies), and 50 mM 2-deoxyglucose (Sigma-Aldrich). The mitochondrial stress test was performed in glucose-containing media with sequential injections of 1 μM oligomycin, 1 μM FCCP, and 0.5 μM antimycin A/rotenone (Agilent Technologies).

### Reactive oxygen species production

Cells were incubated with 5 μM DCFH-DA (Sigma, D6883) or 5 μM MitoSOX™ (Invitrogen, M36008) for 30 minutes at 37 °C, followed by 1 μM N-formyl-Met-Leu-Phe (fMLP; Sigma-Aldrich, F3506) stimulation. Fluorescence was measured at baseline and 30 minutes (BioTek, 485/535 nm for DCFH-DA, 510/580 nm for MitoSOX). Notably, DCFH-DA is a cell-permeable chemical probe used to detect intracellular reactive oxygen species (ROS).

### Respiratory burst measurements

Respiratory burst was assessed using the Respiratory Burst Assay Kit (Cayman Chemical) following the manufacturer’s instructions. Briefly, BMDMs were incubated with dihydrorhodamine-123 for 1 h at 37 °C. Rhodamine-123 fluorescence intensity was determined using the FL1 channel (green laser) of the Accuri C6 flow cytometer.

### Isotopic tracing-based metabolic flux analysis

BMDMs were incubated for 24 h in glucose-free RPMI supplemented with 10% FBS and 11 mM [1,2-^13^C_2_] D-Glucose- (Sigma-Aldrich 453188). Cells (1–3 ×10^6^) were then washed with cold PBS, and metabolites were extracted in methanol containing norvaline (internal standard), chloroform and water. After vortexing (30 min, 4 °C) and centrifugation (1,000 ×g, 20 min, 0 °C), aqueous and organic phases were separated, dried, and stored at −80 °C. Polar metabolites were derivatized with MBTSTFA + 1% TBDMCS (Thermo Fisher) and analyzed by GC-MS (Agilent 7890A/5977C, 30-m HP-5 MS column). Lactate isotopologue peaks were integrated using the MATLAB-based PIRAMID software (55), and the integrated ion counts were normalized to the protein content of the cell extract samples.

### Western blot

Cells were lysed in RIPA buffer (Sigma) with phosphatase and protease inhibitor (Sigma). Protein levels were quantified by BCA assay (Thermo Scientific). 20 μg samples were denatured in 2×Laemmli buffer, heated at 95 °C for 8 minutes, separated on 4–20% Tris-glycine gels (Bio-Rad), and transferred to nitrocellulose membranes. Membranes were blocked with 5% BSA/TBST (1 hour), incubated overnight (4 °C) with primary antibody against gp91phox (Santa Cruz, sc-130543, 1:1000), p47phox (Santa Cruz, sc-17845, 1:500), P-p47phox (pSer345) (Sigma, SAB4504721, 1:500), p40phox (Santa Cruz, sc-48388, 1:500), SOD-2 (Santa Cruz, sc-133134, 1:1000), HO-1 (Santa Cruz, sc-136960, 1:1000) followed by HRP-conjugated secondary antibody (Cell Signaling Technology, 7074, 7076). Protein bands were visualized using Clarity Max ECL substrate (Bio-Rad) and HyBlot-ES film (Thomas Scientific), and band intensities were quantified using ImageJ (NIH).

### NADP+/NADPH quantification

NADP^+^/NADPH levels were quantified from cell lysates using the NADP^+^/NADPH Assay Kit (Sigma, MAK479) according to the manufacturer’s protocol. Absorbance was measured at 565 nm using a BioTek ELx800 plate reader.

### GSH/GSSG quantification

GSH and GSSG levels were measured using the GSH/GSSG-Glo™ Assay (Promega, V6611) according to the manufacturer’s protocol.

### ATP quantification

Cells (5 ×10^5^) were lysed with Luciferase Cell Culture Lysis Reagent (Promega). ATP levels were measured using a luciferin-luciferase–based ATP assay (Invitrogen, A22066) according to the manufacturer’s instructions. Luminescence was measured using a BioTek ELx800 plate reader.

### Nitrite quantification

Nitrite levels in cell culture supernatants (1 ×10^6^) were quantified using the Griess Reagent System (Promega, G2930) according to the manufacturer’s instructions. Absorbance was measured at 520 nm.

### Xanthine oxidase activity measurement

XO activity was measured using the Xanthine Oxidase Assay Kit (Sigma, MAK497) per the manufacturer’s protocol. Fluorescence was recorded at Ex = 530 nm/Em = 585 nm.

### RNA-seq analysis

Total RNA was extracted using the RNeasy Mini Kit (Qiagen). RNA quality and concentration were evaluated using a NanoDrop 2000 spectrophotometer (Thermo Scientific). Sequencing and downstream analyses were conducted by Novogene with 3 biological replicates per condition (California, U.S., www.novogene.com). The data discussed in this publication have been deposited in NCBI’s Gene Expression Omnibus (56) and are accessible through GEO Series accession number (GSE278668).

### Phagocytosis and killing assay

BMDMs (1 ×10^5^ cells/well) were plated in 96-well tissue culture plates. *P. aeruginosa* was washed, passed through a 26 G needle, and diluted to 1 ×10^7^ CFU/mL in RPMI 1640 with 0.1% FBS. Cells were washed with PBS and inoculated with 100 μL of bacterial suspension, centrifuged (515 ×g, 4 minutes, 4 °C) and incubated at 37 °C for 11 minutes. Subsequently, gentamicin (300 μg/mL) was added for 15 min, followed by PBS washes. For immediate bacterial load, cells were lysed (0.02% Triton X-100) and plated to count CFU. For the killing phase, media was replaced, and cells were incubated for 3 h before lysis and CFU enumeration (13). The number of *P. aeruginosa* was determined by subtracting the final bacterial load after 3 hours of incubation from the initial load measured at 15 minutes post-infection.

### Statistical analysis

Unless stated otherwise, statistical analyses were performed using GraphPad Prism version 10.2 (GraphPad Software, San Diego, CA). Comparisons between two groups utilized two-tail Student’s T-test. One-way ANOVA with Tukey’s *post hoc* test was used when comparing multiple groups under a single factor. When experiments involved two independent factors with equal sizes, Two-way ANOVA with Tukey’s *post hoc* test was applied. For unequal group sizes, a linear mixed-effects model was used with genotype and treatment as fixed effects and experiment as a random effect. Statistical significance was defined as p ≤ 0.05. Gene expression analysis methods are detailed above. Unless stated otherwise, data are presented as mean ± SEM.

## Data Availability

The datasets presented in this study can be found in online repositories. The names of the repository/repositories and accession number(s) can be found below: https://www.ncbi.nlm.nih.gov/, GSE278668.
