# Supplementary material for: Monophosphoryl lipid A boosts macrophage antimicrobial immunity by metabolically regulating source-specific ROS generation
Source: Front Immunol. 2026 Mar 30;17:1745195. doi: 10.3389/fimmu.2026.1745195 (PMC13070754; doi:10.3389/fimmu.2026.1745195)
Supplement: Supplementary file 1 [file DataSheet1.pdf]

SUPPLEMENTAL FIGURES

Monophosphoryl lipid A boosts macrophage antimicrobial immunity through metabolically regulating source-specific ROS generation.

Dan. Hao[1], Benjamin D. Klein[1], Margaret A. McBride[2], Julia K. Bohannon[1,2], Naeem K. Patil[1], Xenia D. Davis[1], Mary A. Oliver[2], Mei Lin Ning. Dye[3]. Sara. Weidenbach[4], Jamey D. Young[4,5], Edward R. Sherwood[1,2,3*]

1. Department of Anesthesiology, Vanderbilt University Medical Center, 1211 Medical Center Drive, Nashville, Tennessee 37232, United States

2. Department of Pathology, Microbiology and Immunology, Vanderbilt University Medical Center, 1211 Medical Center Drive, Nashville, Tennessee 37232, United States

3. East Tennessee State University, Quillen College of Medicine, Johnson City, Tennessee 37614, United States

4. Department of Chemical and Biomolecular Engineering, Vanderbilt University, Nashville, Tennessee

5. Department of Molecular Physiology and Biophysics, Vanderbilt University, Nashville, Tennessee

* Corresponding author: Department of Pathology, Microbiology and Immunology, Vanderbilt University Medical Center, 1211 Medical Center Drive, Nashville, TN 37232, United States. Email: edward.r.sherwood@vumc.org.

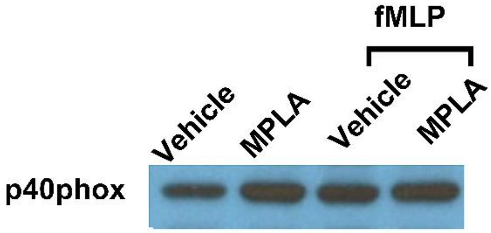

Supplemental Figure 1. Representative immunoblot of p40^{phox} in vehicle- or MPLA- treated BMDMs with/without fMLP restimulation. Bone marrow-derived macrophages were incubated with vehicle or MPLA (1 μg/ml) for 24h, followed by restimulation with 1 μM N-formyl-Met-Leu-Phe (fMLP) for 30 minutes.

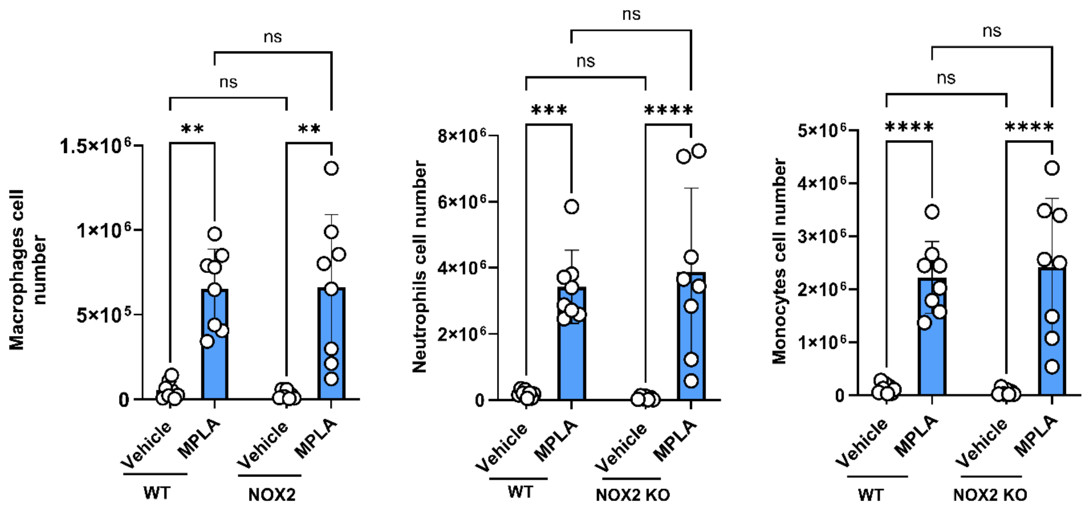

Supplemental Figure 2. Leukocyte number in the peritoneal lavage in wild-type (WT) and NOX2-deficient (KO) mice treated with vehicle or MPLA following P. aeruginosa infection. Wild-type (WT) and NOX2 knockout (KO) mice were administered MPLA (20 μg) on two consecutive days. Twenty-four hours after the final MPLA dose, mice were challenged with *Pseudomonas aeruginosa* for 6 hours. Macrophage, neutrophil, and monocyte populations in the peritoneal lavage were quantified by flow cytometry.

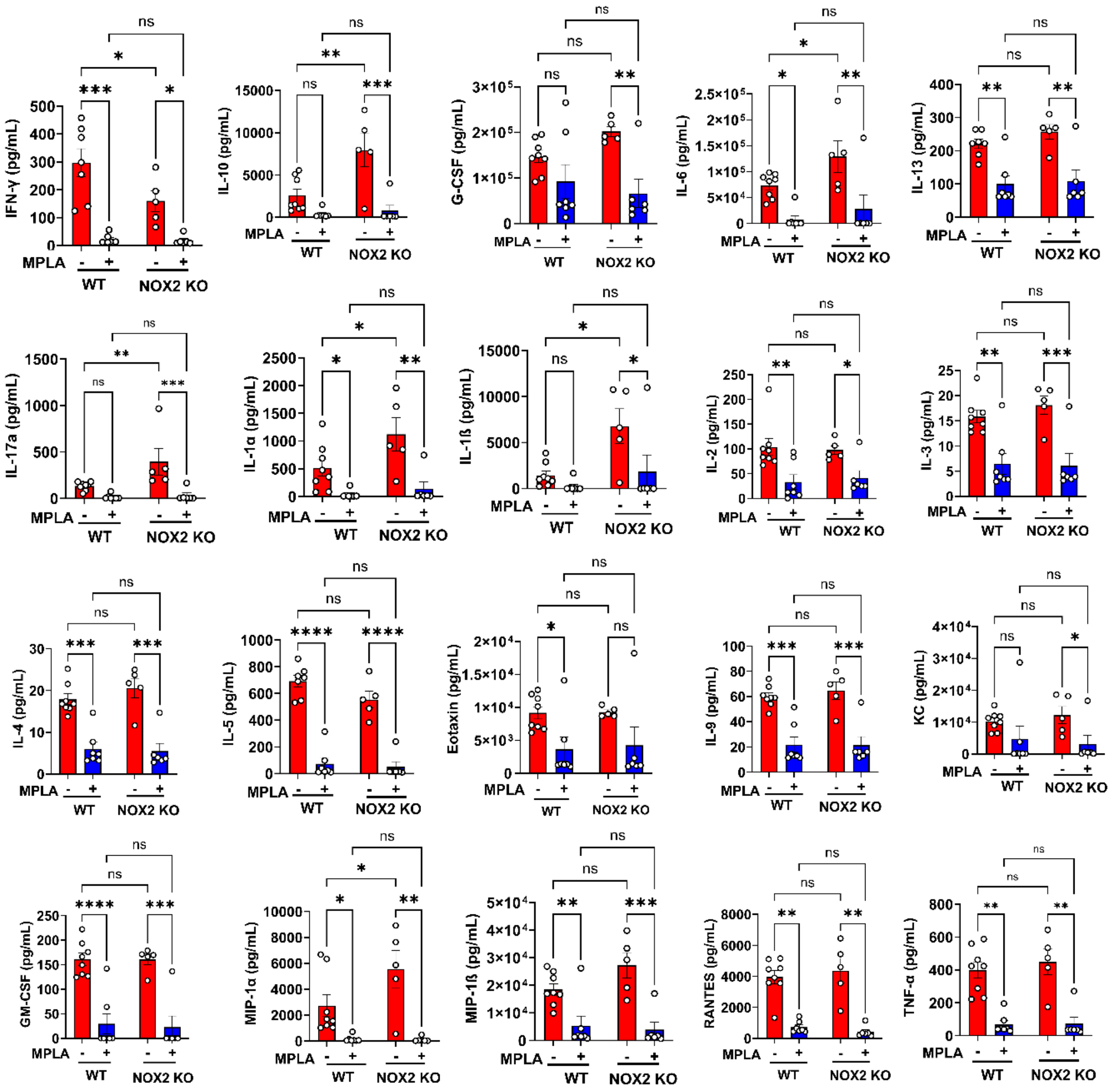

Supplemental Figure 3. Cytokine panel analysis in wild-type (WT) and NOX2-deficient (KO) mice treated with vehicle or MPLA following P. aeruginosa infection. Wild-type (WT) and NOX2 knockout (KO) mice were administered MPLA (20 μg) on two consecutive days. Twenty-four hours after the final MPLA dose, mice were challenged with *Pseudomonas aeruginosa* for 6 hours. (A – C) cell number of macrophages, neutrophils and monocytes in peritoneal lavage. (D – X) Plasma cytokine levels. N = 5–8 per group. Data are presented as mean ± SEM. Statistical significance was assessed using mixed-model analysis.
